# Operationalizing and Measuring Informed Choice in Health Care: An Umbrella Review

**DOI:** 10.1177/0272989X251413276

**Published:** 2026-03-15

**Authors:** Holly Sprosen, Chiara Re, Grant D. Stewart, Juliet A. Usher-Smith

**Affiliations:** General Surgery Department, Middlemore Hospital, Auckland, New Zealand; Department of Public Health and Primary Care, University of Cambridge, Cambridge, UK; Department of Surgery, University of Cambridge, Cambridge Biomedical Centre, Cambridge, UK; IRCCS San Raffaele Hospital, Unit of Urology, Vita-Salute San Raffaele University, Milan, Italy; Department of Surgery, University of Cambridge, Cambridge Biomedical Centre, Cambridge, UK; CRUK Cambridge Centre, University of Cambridge, Cambridge Biomedical Centre, Cambridge, UK; Department of Public Health and Primary Care, University of Cambridge, Cambridge, UK

**Keywords:** informed choice, informed consent, umbrella review, psychometrics, systematic reviews as topic, health services, measurement instruments

## Abstract

**Background:**

Informed choice is of the highest importance in health care. However, confusion and challenges remain toward how it is conceptualized and measured.

**Purpose:**

This umbrella review aimed to establish how informed choice is operationalized in health care and the characteristics and performance of the most commonly used measurement instruments.

**Data Sources:**

Four electronic databases (Ovid MEDLINE, Ovid EMBASE, APA PsycINFO, and Cochrane Library) were searched up to January 29, 2024. Reference lists of included studies were hand searched for further relevant publications.

**Study Selection:**

After the titles and abstracts of 10,434 articles were screened by one reviewer and 10% were screened by a second reviewer for consistency, 2 reviewers independently screened 60 full-text articles for inclusion. Key eligibility criteria included systematic reviews in adult health care settings where the aim included an evaluation of measures of informed choice. Sixteen articles were included.

**Data Extraction:**

Data were independently extracted by 2 reviewers using a standardized template. *Data Synthesis.* Data were synthesized using the summarization technique with systematic reviews as the main unit of analysis and additional subanalysis of primary measurement instruments identified.

**Limitations:**

Heterogeneous definitions complicate search strategies, and eligibility criteria may limit external validity. The ROBIS appraisal identified many reviews as high risk of bias, limiting the conclusions drawn. Due to heterogeneity, meta-analysis was not possible, and conclusions were limited to narrative reviews.

**Conclusions:**

There remains no consensus on how informed choice should be conceptualized and measured within health care. This review attempts to bridge these gaps by presenting available concepts and instruments for clinicians, researchers, and policy makers. Future recommendations include achieving consistent definitions of informed choice and related concepts, followed by the use of standardized, validated, multidimensional instruments informed by theory in diverse populations.

**Highlights:**

- Informed choice is of key importance and increasingly emphasized across health care.
- Despite this importance, confusion and challenges remain regarding how informed choice is conceptualized and measured in health care.
- Consistent definitions and the use of standardized, validated, multidimensional instruments, informed by theory and patients themselves, in diverse populations should be the first steps to improve this.
- These recommendations apply to all in health care, including health professionals, researchers, and policy makers.

Informed choice is of the highest importance in health care and increasingly emphasized, especially in clinical trials, treatment decisions and screening.^1–5^ In addition to the ethical and legal responsibilities,^6–8^ informed choice carries potential economic and patient outcome benefits.^9,10^ Measuring informed choice, however, remains difficult,^11–13^ including a lack of consensus on what qualifies as “informed”^14–17^ and individual variability.^6,14,18,19^ Evidence shows that patients’ needs for information differ.^11,15,16,20,21–23^ One surgical-based trial found that 88% of patients wanted to know the short-term effect on activities postoperatively, yet only 62% wanted the technical details.^24^ Similarly, in breast screening, a qualitative study found disagreement among participants as to whether they would want to be informed of overdiagnosis as a risk.^25^ What constitutes necessary information by those responsible for imparting it—health professionals, researchers, or policy makers—can also differ from the public’s wants.^11,14,15,20,21^ A separate UK study on breast screening leaflets found that while experts advocated for scientific precision and detailed statistics, participants favored simple explanations.^26^

Another challenge is the overlapping concepts in literature and guidelines.^11,15,20,27^ Table 1 presents example definitions for informed choice and related concepts. As observed, O’Connor et al.’s definition of an effective decision being “informed, consistent with values and congruent with behaviour”^28^ has also formed the basis for concepts including informed choice, informed decision, and decision quality.^15,20,27–31^

**Table 1:** Example Definitions of Informed Choice and Similar Concepts

Concept	Definition
Informed choice	“. . . based on relevant knowledge, consistent with the decision-maker’s values and behaviourally implemented.”^20,28^
Informed consent	“. . . (1) competence, (2) disclosure, (3) understanding, (4) voluntariness and (5) consent.”^32^
Informed decision	“. . . patients (1) are aware of relevant alternatives and their outcomes, (2) have clarified expectations of outcomes that are reasonably aligned with reality, and (3) are aware of the nature of the conflict in the decision.”^28^
Informed decision making	“. . . individual’s overall process of gathering relevant health information from both his or her clinician and from other clinical and nonclinical sources with or without independent clarification of values.”^33^
Effective decision	“. . . informed, consistent with values and congruent with behaviour.”^28^
Decision quality and process	“. . . decision-making process attributes (i.e., recognise decision, feel informed, clear values, discuss goals with health care provider, be involved) or the two decision quality attributes (i.e., knowledge (including realistic expectations), and concordance).”^18^
Evidence informed patient choice	“. . . providing people with research-based information about the effectiveness of healthcare options and promoting their involvement in decisions about their treatment.”^34^

Subject experts have attempted to distinguish between terms.^15,30,35^ Jepson et al.^15^ argued that informed choice, unlike informed consent, is more suitable in screening where individuals are invited and choose to participate, without necessarily interacting with health care professionals. Østerlie et al.^14^ agreed, seeing informed choice as fitting for nonobligatory health services. Rimer et al.^35^ went further to differentiate informed consent from informed or shared decision making. They emphasized decision making’s focus on value congruence (an individual’s values reflected in their decision) and patients having flexible participation. Briss et al.^30^ also distinguished shared from informed decision making, noting that shared decision making involves both patients and professionals, often in a clinical setting, participating in decisions in a personalized manner. They further suggested that informed decision making can occur without professional involvement, such as in screening contexts.^30^ While these explanations clarify subtle differences, confusion remains over when each concept is more appropriate.^14^

This confusion extends to which components should be measured.^27,36–38^ Knowledge, representing “informed,” appears logical to measure,^11,15,30,31^ yet other domains, such as anxiety during decision making, are more contentious.^23,19,31,39^ Bekker et al.^31,39^ cautioned against the use of anxiety when measuring the effectiveness of patient decision aids (PtDAs)—tools designed to support an individual’s decision process to reach an informed choice^40,41^—finding it inadequate and lacking evidence in its association with adverse consequences of decision making.^39^ Despite this, the 2017 Cochrane review of PtDAs found that 30% of studies continue to assess anxiety.^42^

Despite existing guidelines to support the choice and development of measuring instruments,^43^ such as the COnsensus-based Standards for the selection of health Measurement INstruments (COSMIN),^44–46^ or the International Patient Decision Aid Standards (IPDAS) collaboration,^47^ and more specific Core Outcome Sets, including Gillies et al.^48^ for informed consent in randomized control trials (RCTs), or Convie et al.’s^49^ evaluation of surgical informed interventions, measuring informed choice remains difficult.^11,13,19^ Systematic reviews provide some insight,^11,13,19^ although they are mostly limited to the comparison of one concept across individual studies.^50^ Furthermore, accessing and using high-quality evidence among multiple reviews is difficult.^51^ An umbrella review, which collates systematic reviews, is an efficient way to gather the best available evidence and is often used in health care policy.^50,52–54^ The approach is especially useful for bridging gaps between closely related concepts often analyzed separately, enhancing the understanding of different perspectives.^50,51,55^

This umbrella review aims to establish how informed choice is operationalized and measured across health care and to determine the characteristics and performance of the most commonly used or appraised measurement instruments identified for informed choice. This will enable health professionals, researchers, and policy makers alike to select the concept or instrument that best suits their work and support future high-quality research in the field.

## Methods

Methods were guided by the Joanna Briggs Institute Umbrella Review Approach and Cochrane’s Overview of Reviews.^50,52^ Reporting adheres to the Preferred Reporting Items for Overviews of Reviews (PRIOR).^53^ The protocol for this review was registered with the International Prospective Register of Systematic Reviews (PROSPERO CRD42024513810).^56^

### Search Strategy

Search strategies were devised in consultation with medical librarians. The following databases were searched for relevant studies on January 29, 2024.

- Ovid MEDLINE (1946 to January 26, 2024)
- Ovid EMBASE (1974 to January 26, 2024)
- APA PsycINFO (ProQuest)
- Cochrane Library (Advanced Search)

Searches were limited to English language, human studies, meta-analysis or systematic review, and 1990–current, reflecting the limited number of systematic reviews before 1990.^50^ The search strategy exemplar for Ovid EMBASE is presented in Appendix 1. The reference lists of the included studies were hand searched to identify further relevant publications.

### Eligibility Criteria

The inclusion and exclusion criteria applied are shown in Table 2. For consistency and conciseness in this review, when referring to measures of informed choice, it will encompass measures of all the concepts stated in Table 3 unless explicitly stated otherwise.

**Table 2:** Eligibility Criteria

SPIDER	Inclusion Criteria	Exclusion Criteria
S – Sample	Reviews based in human health care settings Reviews on adult populations (age 18 y and older) with presumed capacity/competency Reviews assessing measures from the patient perspective	Reviews based in non–health care settings Reviews on pediatric populations (age <18 y) Reviews when choice is made by proxy/surrogate individuals Reviews in health care settings when an individual’s capacity/competency is diminished or in question Reviews on populations whose mental health and the influence of this on informed choice is the focus of the review Reviews in which the patient perspective was not considered in measure, e.g., from health care professional or observer only
PI – Phenomenon of Interest	The main aim(s) of the review include an evaluation of measures of informed choice and its counterparts outlined in Table 3	Review aim does not include an evaluation of measures of informed choice; for example, the aim is to evaluate interventions or experiences
D – Design	Systematic reviews, as defined by the PRIOR guidelines, will be included^53^; meta-analyses and qualitative evidence synthesis included Reviews can include primary study design of all types Reviews must be published in English language with full-text availability	Other review types including scoping, rapid evidence assessment, evidence, and gap mapping Editorials or opinion pieces Reviews not published in the English language No full-text availability
E – Evaluation	Reviews evaluating measures of informed choice in part or whole as presented in Table 3	Reviews assessing concepts different from those in Table 3
R – Research Type	All methodologies	

**Table 3:** Concepts Relating to Informed Choice

Concept
Informed choice
Informed consent
Informed decision
Informed decision making
Effective decision
Decision quality and process
Evidence-informed patient choice

One reviewer screened all titles and abstracts, with 10% of articles randomly screened by a second reviewer for consistency. Where disagreements on an article’s inclusion for full-text screening arose, these articles were included. Eligibility criteria were then piloted using 10 full-text articles, with final eligibility criteria formalized subsequently with third and fourth reviewers. Two reviewers then applied the criteria to assess all full-text articles independently. Disagreements on final eligibility were resolved with all reviewers, with consensus on included articles reached. In the case of overlapping systematic reviews, all relevant reviews were included.^52^

### Data Extraction

Data extraction was performed independently by 2 reviewers using a standardized template (Appendix 2). Extracted data included review demographics; the concept assessed; definitions; how measurement instruments were evaluated; key study findings, including any psychometric results, specific measurement instruments, and their properties; and any limitations or recommendations made by reviews. Any discrepancies were resolved with third and fourth reviewers, with a consensus reached.

### Critical Appraisal

Independent critical appraisal by 2 reviewers using the Risk Of Bias In Systematic Reviews (ROBIS) tool was conducted.^57^ The third and fourth reviewers were consulted for any disagreements to reach a consensus. All systematic reviews were included to assess the breadth of available evidence, irrespective of appraisal outcomes.

### Data Synthesis

Data synthesis was performed with the systematic reviews as the unit of analysis and then again in a subanalysis with the primary instruments measuring informed choice identified within the systematic reviews as the unit of analysis to meet the 2 aims of the review.

Extracted data from systematic reviews were analyzed and reformatted in alignment with summarization techniques for umbrella reviews.^50,52^ The subanalysis focused on the most commonly used or highly rated measures of informed choice, selected based on their frequency cited in reviews or when singled out for appraisal or discussion by reviews, with additional data from primary studies and wider literature supplementing findings.

Meta-analysis was not performed due to the heterogeneity between reviews and the inclusion of qualitative and quantitative data.

## Results

**Figure 1:**
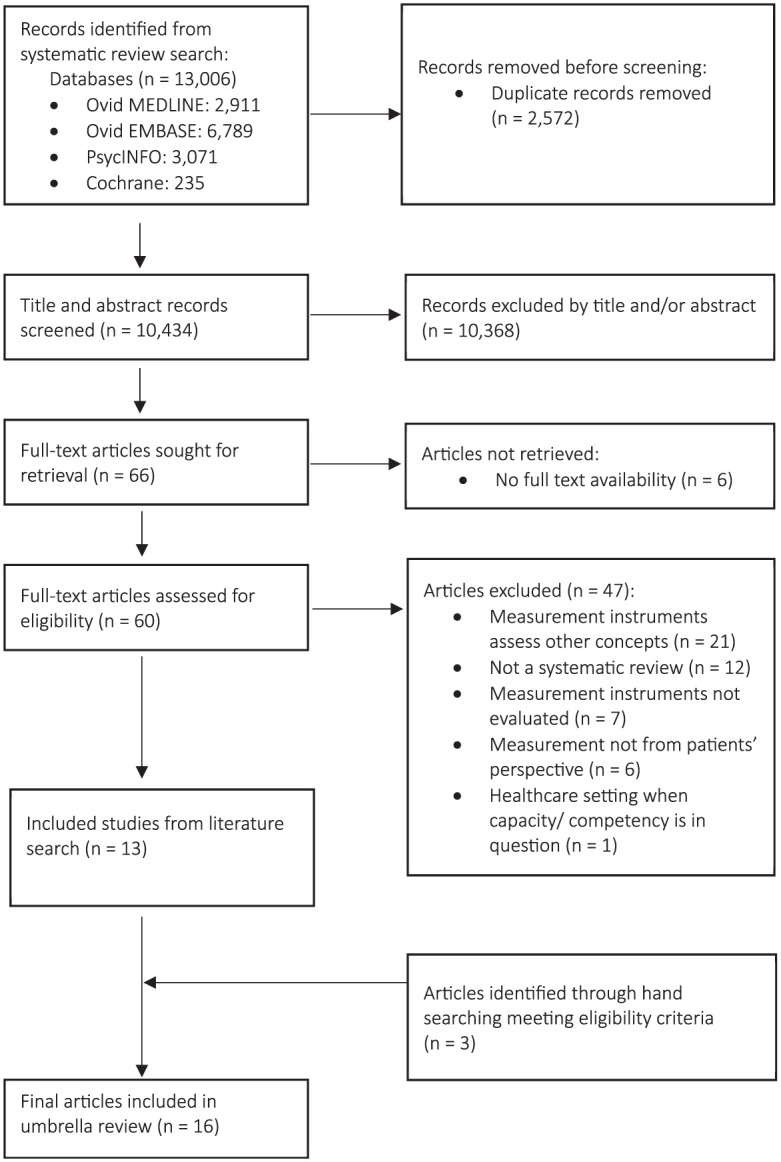
PRIOR flow diagram of systematic review search results based on PRISMA template.^53,58^

The search results are presented in Figure 1. In total, 13,006 citations were identified from electronic databases. A total of 2,572 duplicates were removed, leaving 10,434 unique results. Title and abstract screening resulted in 10,368 exclusions. The consistency of the interrater reliability for screening was 99.45%. Of the 66 remaining articles, 6 had no full-text availability, with the other 60 full texts screened using eligibility criteria. Forty-seven articles were excluded for not meeting the criteria. Appendix 3 provides the list of excluded articles with reasons. Thirteen articles from the electronic search met eligibility criteria, with a further 3 eligible articles identified through hand searching the reference lists. The final 16 systematic reviews are presented in Table 4.

**Table 4:** Demographic and Conceptualization Features of the Included Studies

Reference: Author and Year	Number and Type of Studies Included	Primary or Updated (Formal, Framework, or Extended) Review	Context/Setting	Concept	Number of Measures Identified
Afolabi et al,^36^ 2014	29 Clinical trials (quantitative and qualitative)	Primary	Clinical research studies in sub-Saharan Africa	Comprehension (informed consent)	Not stated
Ames et al,^27^ 2015	33 RCTs and prospective (observational, quasi-experimental, pilot, cross-sectional, and longitudinal)	Primary	Population reproductive genetic screening programme studies	Informed choice	>20
Gillies et al,^37^ 2017	14 RCTs only	Primary	Clinical research studies	Informed consent	14
Kennedy,^19^ 2003	33 RCTs only	Primary	Decision aid studies	Decision aid effectiveness (effective decision)	Not stated
Kryworuchko et al,^59^ 2008	35 RCTs only	Primary	Screening and treatment decisions Cochrane 2003 PtDAs systematic review	Decision quality and decision process	35
Mamotte et al,^60^ 2015	15 Empirical studies (quantitative and qualitative)	Primary	Clinical research studies	Voluntariness (informed consent)	Not stated
Montalvo et al,^61^ 2014	27 RCTs and observational	Primary	Clinical research studies	Comprehension (informed consent)	Not stated
Mullen et al,^23^ 2006	36 Interventional trials, cross-sectional, cohort, and qualitative	Primary	Cancer screening studies	Informed decision making	131
Munro et al,^62^ 2015	18 RCTs only	Updated (framework)^29,63^	Screening and treatment decisions Cochrane 2014 PtDAs systematic review	Value congruence (decision quality)	Not stated
Raper et al,^64^ 2021	70 RCTs only	Primary	Surgical trials	Informed consent	Not stated
Sand et al,^38^ 2010	34 RCTs and observational	Primary	Clinical research studies	Understanding (informed consent)	Not stated
Sepucha et al,^29^ 2010	49 RCTs and observational	Primary	Medical decision-making studies	Value concordance (decision quality)	59
Sepucha et al,^43^ 2014	86 RCTs only	Updated (extended)^59,65^	Screening and treatment decisions Cochrane 2011 PtDAs systematic review	Decision quality and decision process	178
Sherman et al,^21^ 2021	16 Original studies (quantitative only)	Primary	Medical practice studies	Informed consent	16
Trenaman et al,^66^ 2021	49 RCTs only	Updated (formal)^43^	Screening and treatment decisions Cochrane 2014 and 2017 PtDAs systematic review	Decision quality and decision process	109
Winn et al,^63^ 2015	61 RCTs, observational, quasi-experimental, and qualitative	Updated (formal)^29^	Medical decision-making studies	Value concordance (decision quality)	72

PtDA, patient decision aid; RCT, randomized controlled trial.

### Critical Appraisal

The ROBIS results for each review are presented in Appendix 4. Appendix 5 demonstrates the percentage of reviews attaining a low, high, or unclear bias risk for each phase 2 domain and phase 3’s overall risk. Fifteen reviews were rated as being low risk for study eligibility criteria, identification, and selection of studies. One review prompted high-risk assessments for both domains due to a lack of transparent criteria, adequate search strategies, and justification for decisions.^19^ Most studies were rated as high risk for data collection, study appraisal, synthesis, and findings, with 5 rated as low risk. This was largely due to no risk-of-bias assessment performed by the reviews themselves. Of the 5 rated low risk, 1 performed their assessment,^61^ and the remaining used the Cochrane assessment on which they were based.^43,62–66^ This contributed to 10 reviews being scored as high risk of bias overall and 6 reviews being scored as low risk. One review that did not perform a risk-of-bias assessment justified its reasons—the review focused on methods rather than outcomes—and discussed this limitation.^63^ As the concern was addressed, a low risk of bias was given.

### Review Demographics

Review publication dates ranged from 2003^19^ to 2021.^21,66,64^ The number of studies per review ranged from 14 to 86.^37,43^ Seven reviews included only RCTs,^19,37,43,62–66,64^ with the remainder including additional study types.^i^ Three reviews focused on specific contexts—surgical trials,^64^ reproductive genetic screening,^27^ and cancer screening^23^—with the remainder consisting of generalized decision-making and clinical research studies.^ii^ Concepts assessed included informed consent,^21,37,64^ informed choice,^27^ informed decision making,^23^ decision aid effectiveness (effective decision),^19^ and decision quality or process.^43,59,66^ Seven reviews focused on a component of these concepts, including comprehension,^36,61^ understanding,^38^ or voluntariness,^60^ for informed consent and value congruence or concordance as part of decision quality.^29,62,63^ In addition to a narrative synthesis, 2 reviews also performed a meta-analysis using a random effect model due to study heterogeneity.^36,62^ Of the reviews that did not include a meta-analysis, 5 justified this as not possible due to heterogeneity.^27,29,19,61,64^

From the 16 systematic reviews, 15 had available lists of the included primary studies they reviewed.^iii^ In the review by Mullen et al.,^23^ the authors reported their data in a table that was inaccessible online. From the 15 reviews, there were a total of 569 primary studies. This included 456 distinct studies and 113 that were present across more than 1 review, resulting in a primary study overlap of 20%.

### Summary of Review Evidence

Table 5 summarizes common themes identified across systematic reviews regarding how informed choice is operationalized. These themes cover the underlying conceptualization, the methods of measurement (referred to as measurement instrument or tool), and how they are reported on by researchers in the literature.

**Table 5:** Summary of Common Themes on Informed Choice Operationalization across Systematic Reviews

Themes
• Variable definitions and overall conceptualisation of what the instruments are developed to measure, e.g., informed choice^21,23,27,29,19,36–38,43,61–63,60^
• Lack of theoretical framework and patient and public involvement informing instrument development^37,59,63,60^
• Potential bias through the timing and format of the instruments and limitations information gained from certain formats, e.g., questionnaires^29,36,38,61,63,60^
• Heterogeneity and lack of standardization across instruments^21,23,27,29,19,36–38,61,62,63,60^
• Large focus on knowledge and little assessment of other areas, e.g., value congruence^21,23,19,37,62,66,64^
• Lack of psychometric performance including validation of instruments^21,23,27,36,38,43,61,66,60^
• Limitations in reporting in literature^21,23,27,29,19,36–38,43,61–63,60^

### Variable Definitions for the Conceptualization of Informed Choice

Table 6 details the definitions each review provided for informed choice. No 2 definitions for the same concept are identical, with a variety of sources referenced. Some reviews described similar definitions for terminologically different concepts. For example, Ames et al.’s informed choice definition includes “having a factual understanding and making a choice aligned with one’s values.”^27^ Comparatively, Kennedy’s definition of a “good” (effective) decision includes “informed, should agree with the subject’s values and preferences.”^19^ Both share O’Connor et al. as a source^67,68^ and are similar to definitions describing decision quality and informed decision making.^23,29,43,62–63^ Others’ definitions are based on organizational or governing recommendations, for example, the World Medical Association’s (WMA’s) Declaration of Helsinki.^1^ Variability in definitions and conceptualization was a common theme identified in 9 of 16 reviews.^23,27,29,36–38,61,62,60^

**Table 6:** Definitions of the Concepts Measured in Reviews

Concept	Review	Definition	Definition Source
Informed consent	Gillies et al.	“In broad terms, informed consent for research covers aspects such as capacity, disclosure, understanding, voluntariness and permission.” “For consent to be considered valid, in accordance with existing regulatory frameworks, it must be voluntary, informed and with the individual providing consent having sufficient capacity to do so.”	Referenced: WMA 2008^1^ ICH 1996^69^ Beauchamp and Childress 2012^6^
	Raper et al.	“The ethical foundation of informed consent can be traced to the promotion of 2 values: personal well-being and self-determination.” “Yet the process by which patients given informed consent continues to evolve. As evidence, a 2002 charter, promulgated by number of international professional organizations, recognised a third imperative: social justice.”	Quoted: President’s Commission 1982^70^ Referenced: Project of the American Board of Internal Medicine Foundation 2002^71^
	Sherman et al.	“Obtaining patient consent is a necessary and critical process enshrined in medical practice, that is characterised within a model of three domains: (provision of) Information, Comprehension (of information by the patient), and Voluntariness (of the patient’s decision without coercion).”	Referenced: Dennehy and White 2012^72^
	Afolabi et al.	“International ethical guidelines stipulate that informed consent must be given in a comprehensible manner to a competent person who freely decides to participate after understanding the information.”	Referenced: National Bioethics Advisory Commission 2011^73^ Council for International Organisation of Medical Sciences 2002^74^ Marshall 2006^75^
	Montalvo et al.	“US Department of Health and Human Services (HHS, 1998) has articulated the following required elements of informed consent: (a) the purpose of the research; (b) risks and benefits associated with participation; (c) participation is totally voluntary, without coercion; (d) the distinction between research and clinical care; (e) the opportunity to ask questions; and (f) alternatives to participation.”	Referenced: United States Department of Health and Human Services 1998^76^
	Sand et al.	“The consent process generally includes disclosing verbal and written information explaining the purpose of the trial, procedures, possible risks and benefits, source of finance, potential conflicts of interest and the researchers’ institutional affiliation (as described in the Declaration of Helsinki [World Medical Association 2008]). However, a truly informed consent presupposes not only the disclosure of the required information, but also that the subject understands it.”	Referenced: WMA 2008^1^
Voluntariness (informed consent)	Mamotte et al.	“if he or she wills the action without being under the control of another’s influence.” (Voluntariness) “a process by which an individual voluntarily expresses his or her willingness to participate in a particular trial, after having been informed of all aspects of the trial that are relevant to the decision to participate.” (Informed consent)	Quoted: Beauchamp and Childress 2009^32^ ICH 1996^69^
Informed choice	Ames et al.	“Definitions of informed choice more commonly involve at least two elements: having a factual understanding and making a choice aligned with one’s values.” “Some definitions distinguish between choice and decision making with the inclusion of deliberation and the behavioural implementation of the decision to reflect the cognitive processes involved in decision making. However, underlying these various definitions is the same concept of an informed and autonomous choice.”	Referenced: Marteau et al. 2001^20^ Van der Berg et al. 2005^77^ Bekker et al. 1999^31^ O’Connor 1995^67^ Jepson et al. 2005^15^
Informed decision making	Mullen et al.	“Informed decision-making is generally defined as the process that patients go through to make a decision about engaging in a medical or health-related procedure or activity considering benefits, harms, risks, health improvements, the match between these properties and personal values and preferences and understanding the uncertainty and limitations of the procedures.”	Referenced: Briss et al. 2004^30^
“Good” decision (effective decision)	Kennedy	“A commonly employed definition of what constitutes a good decision, proposed by O’Connor et al. is that a decision should be informed, should agree with the subject’s values and should be implemented.” (Effective decision) *No definition given for decision-aid effectiveness*	Referenced: O’Connor et al. 1998^68^
Decision quality and decision process	Kryworuchko et al.	“In addition to evaluating decision quality, that is the extent to which decisions were informed and congruent with values, the International Patient Decision Aids Standards (IPDAS) Collaboration identified decision process criteria that can establish the effectiveness of decision aids. These include: awareness that a decision needs to be made, knowledge of options and their features, awareness that values affect the decision, being clear about the option features that matter most, opportunity to explore their values and attitudes with health providers, and becoming involved in the decision making process in preferred ways.”	Referenced: Entwistle et al. 2007^78^ Elwyn 2006^41^
	Sepucha et al.	“Decision quality includes sub domains of decision-specific knowledge, realistic expectations and value concordance (or extent to which treatments match patient’s goals). Decision process measures include sub domains of recognition of a decision; feeling informed about options and outcomes; feeling clear about what matters most; discussing the goals of treatment with providers; and being involved in decision making.”	Referenced: Elwyn 2006^41^ Sepucha et al. 2013^18^
	Trenaman et al.	“Extent to which a patient’s eventual choice is informed and consistent with their values.” (Decision quality) “The extent to which PtDA helps patients to recognise that a decision needs to be made; feel informed about the options; be clear about what matters most to them in this decision; discuss goals, concerns, and preferences with their health care providers; and be involved in decision making.“ (Decision process)	Referenced: Elwyn 2006^41^ Sepucha et al. 2013^18^
Value-congruence (decision quality)	Munro et al.	“Match between the chosen option and the patient’s values.”	Referenced: Sepucha et al. 2013^18^
	Sepucha et al.	“Quality of a decision should be measured by the extent to which choices reflect the preferences of well-informed patients and are implemented. This definition requires assessment of 1) how informed patients are, 2) what patients’ preferences are and 3) the patients’ choice of treatment and the treatment implemented.”	Referenced: Hammond et al. 1999^79^ O’Connor et al. 1998^68^ Ratliff et al. 1999^80^ Kennedy 2003^19^ Briss et al. 2004^30^ Sepucha et al. 2004^129^
	Winn et al.	“Another core element of decision quality is concerned with value concordance, or how well the treatment aligns with the patient’s goals and preferences.” “Specifically, value concordance was defined as the association between patients’ preferences concerning health outcomes and/ or medical treatments, and treatment intention or treatment undergone.”	Referenced: Wexler 2012^130^ Sepucha and Ozanne 2010^29^

### Underlying Theories and Patient and Public Involvement for Measure Development

Three reviews examined the role of theory in an instrument’s development. They highlighted how theory clarifies definitions, connects to broader knowledge, and supports construct validity.^37,63^ Gillies et al.^37^ found that only 5 of 14 studies cited a theory, while Sepucha and Ozanne^29^ reported more than half (28/49) referenced a framework, although Winn et al.’s^63^ updated review found fewer (23/61). Two further reviews identified a lack of patient and public involvement (PPI) in an instrument’s development, reported in 5 of 14 and 1 of 8 studies, respectively.^37,59^

### Timing and Format of Instruments

Eleven reviews assessed timing and/or instrument format. Timing varied from immediately after consent to more than a year later.^36–38,60^ Questionnaires with closed-ended questions were most common, while in-depth interviews and focus groups were less frequently used. For example, Mamotte and Wassernaar^60^ found that 12 of 15 studies used questionnaires versus 3 of 15 using interviews. Some reviews used eligibility-mandated questionnaires or excluded purely qualitative studies.^21,27,37^ Six reviews acknowledged the potential bias that may arise through different instrument timings and formats and/or the limitations in information gained from certain formats, for example, questionnaires.^29,36,38,61,63,60^

### Instrument Heterogeneity, Focus on Knowledge, and Concept Evaluation by Reviews

Heterogeneity and the lack of measurement instrument standardization were the most commonly identified themes across reviews (12/16).^iv^ This will be exemplified in later results, although it is reflected in the reviews evaluating them also. Although most reviews examined which concept domains an instrument measures, their methods varied. For example, Sepucha et al. and Trenaman et al. used IPDAS criteria to code decision quality and process,^41,43,66^ while Raper et al. evaluated informed consent through autonomy, beneficence, and social justice.^64,71,81^ Due to the varied concepts and evaluation methods, pooling of the data was not feasible. Instead, Tables 7 to 9 describe the domains assessed by reviews and individual studies within them. The tables are divided based on the definition and concept they assess for comparison. Table 7 covers informed consent reviews; Table 8 shows informed choice, decision making, and effective decision, decision quality, and process; and Table 9 lists specific subdomains related to value congruence.

**Table 7:** Domains of Informed Consent Assessed by Each Review Measuring This Concept and the Proportion of Individual Studies within the Review That Studied Each Domain where Available

Review	Information Provided	Comprehension/Understanding	Voluntariness	Other	Additional Information on Domains Assessed
Afolabi et al.		√			Within understanding most common domains: Generic: right to withdrawal (13/29 studies) Trial specific: study purpose (17/29 studies)
Gillies et al.	√		√	√	Overall most common domains: • Knowledge/understanding (156/179 measures) • Voluntariness (9/179 measure items) • Capacity/competency (14/179 measures)
Mamotte et al.			√		Within voluntariness most common domains: • Freedom to withdraw (7/15 studies) • Freedom to choose to participate (7/15 studies) • Unspecified influences (7/15 studies)
Montalvo et al.		√			Not assessed
Raper et al.		√		√	Overall most common domains: • Comprehension (65/70 studies) • Satisfaction (33/70 studies) • Mental state (30/70 studies)
Sand et al.		√			Within understanding: • General research • Trial-specific information
Sherman et al.	√	√	√		Overall most common domains: • Information (9/16 studies) • Comprehension (7/16 studies) • Voluntariness (5/16 studies)

**Table 8:** Domains of Informed Choice or Decision Making, Decision Quality/Process, or Effective Decision and the Proportion of Individual Studies within the Reviews Measuring Each Domain where Available

Review	Decision Process	Decision Quality			Other Decision Measures
		Knowledge (Informed)	Value Congruence	Other	
Ames et al.	√ 25/33	√ 33/33	√ 17/33	√ Attitudes only (values): 5/33	
Kennedy	√ 18/33	√ 20/33^a^	√ 1/33	√ Attitudes only (values): 1/33	√ Effect on mental state: 1/33
Kryworuchko et al.	√ 13/26	√ 8/26	√ 1/26	√ Realistic expectations: 3/26	√ Choice or adherence to choice: 20/26
Mullen et al.	√ >11/36^b^	√ 27/36	√ 1/36	√ Attitudes (values): 3/36^a^	√ Choice (including intention): 30/36 Perceived cancer threat: 10/36
Sepucha et al.	√ 61/86	√ 60/86	√ 13/86		√ Not specified: 10/86
Trenaman et al.	√ 38/49	√ 36/49	√ 12/49		√ Not specified: 5/49

a Review grouped knowledge and beliefs.

b With table access unavailable, >11 unique studies were calculated from the text.

**Table 9:** Subdomains within Value Congruence from 3 Reviews Solely Assessing This Domain^a^

Review	Value/Preference Method			Choice Method			Value Concordance Method			Knowledge	Informed Decision
	Preference Calculated via Model or Score	Preferences towards Health Outcomes and/or Attributes	Treatment Preference Calculated Directly	Screening/Treatment Undergone	Preferred Screening/Treatment (Intention)	Other	Match	Regression	Other	(Assessed by Studies)	(Assessed by Studies)
Munro et al.	5%	89%	5%	17%	72%	11%	6%	17%	MMIC: 44% Other method or not stated: 33%	Not assessed	Not assessed
Sepucha et al.	43%	32%	25%	80%	19%	1%	49%	44%	1%	27%	4%
Winn et al.	6%	70%	24%	56%	43%	1%	49%	33%	18%	49%	20%

MMIC, Multidimensional Measure of Informed Choice.^18^

a Reported as percentages due to inconsistencies in value reporting as the proportion of measures or individual studies.

Key informed consent domains identified in Table 7 reviews included information provision, comprehension/understanding, voluntariness, and other.^21,36–38,61,64,60^ Within this, knowledge (information provision and/or comprehension) was the most measured in 6 of 7 reviews.^21,36–38,61,64^ One review by Gillies et al.^37^ found knowledge included in 156 of 179 instrument items. Other domains, such as satisfaction, were studied less frequently.^21,37,64^ In specific comprehension/understanding or voluntariness reviews, study purpose and the right to withdraw were measured more than blinding, randomization, or therapeutic misconception.^36,38,60^

Knowledge remained one of the most measured domains in Table 8,^23,27,19,43,66^ although some reviews identified decision process measures such as decisional conflict or role preference^18^ as more popular.^43,59,66^ Despite it being a requirement in the definitions for these concepts, value congruence was less commonly assessed.^23,27,19,43,59,66^

Table 9 reviews all used Sepucha et al.’s framework to define and calculate value congruence and its relationship to knowledge.^29,62,63^ Through this, they identified the variability in individual study methods, drawing conflicting conclusions in some areas.^29,62,63^ Relating to knowledge above, less than half of the individual studies assessed this alongside value congruence,^29,63^ with even fewer reporting if an overall informed decision was made.^29,63^ The Multidimensional Measure of Informed Choice (MMIC),^20^ used in 44% of articles in Munro et al.’s review,^62^ will be discussed further under characteristics of common measurement instruments.

### Psychometric and Other Performance

Seven reviews evaluated the appropriateness, psychometric performance, and clinical sensibility of measurement instruments or their reporting.^21,23,27,43,59,66,60^ Kryworuchko et al. and Sherman et al. appraised measurement instruments themselves using published criteria.^21,41,59,82,83^ Kryworuchko et al.^59^ found all 8 instruments rated positively for feasibility, followed by validity (7/18) and precision the least (1/8). Sherman et al.^21^ found all 16 instruments internally consistent, but divergent (construct) validity was the least met (1/16). Sepucha et al.^43^ and Trenaman et al.^66^ focused on reporting, with reliability and validity reported in 21% to 23% and 6% to 16% of studies, respectively. Although feasibility was appraised positively above,^59^ only 1 study in these reviews reported it.^43,66^ The remaining reviews discussed the number of studies attempting to establish validation and re-reported rather than reassessed these numbers.^23,27,60^ All except 1 of these 7 reviews commented on the lack of validation (or its reporting) of the measurement instruments.^21,23,28,43,66,60^ A further 3 reviews did not evaluate instrument psychometrics to the same extent, although they also commented on the lack of validation of chosen instruments.^36,38,61^

### Limitations in Reporting in the Literature

In addition to the challenges in measuring informed choice, there are those relating to its reporting in research. Table 10 summarizes the common limitations identified when reporting on informed choice measurement. The most common included the decision by investigators to use newly developed measures rather than existing validated instruments (10/16 reviews) and, related to this, the lack of ability to compare between studies due to heterogeneity (10/16).

**Table 10:** Limitations in Reporting on Informed Choice Measurement

Reporting in the Literature
• Decision to use new investigator-developed measures rather than existing validated instruments^36,38,61–59,60^ • Lack of diversity in the population measurement instruments are evaluated^21,23,27,36^ • Scarcity of information on the development and psychometric performance of the instruments reported by researchers^23,27,29,36,38,43,62–66,60^ • When psychometric or clinical sensibility is reported, some metrics are reported more frequently than others are^23,43,66^ • Lack of ability to compare between studies (including meta-analyses) due to heterogeneity in conceptualization and instruments^27,29,19,36–38,43,61,62,63^

### Characteristics of Common Measurement Instruments

Identifying common measures was challenging due to the heterogeneity in how reviews presented and assessed measurement instruments, with some reviews listing only 1 instrument per study^21,37^ and others not naming specific instruments.^36,60^ Tables 11 and 12 highlight the most common measures reviews identified and their features. Psychometric properties were supplemented by additional sources.

**Table 11:** Key Features of Common Measurement Instruments Identified in Systematic Reviews

Measurement Instrument	Concept, Definition, Theory, or Guideline Basis	Format	Scoring	Sample Item
Decisional Conflict Scale (DCS) O’Connor 1995,^67^	Decision conflict/uncertainty “Decisional conflict is a state of uncertainty about the course of action to take.”^67^ Framework derived from Janis et al. and refined by North American Nursing Diagnosis Association.^84,85^	Four versions exist: One for clinical practice (SURE test)^86^ and 3 for research (statement, question, and low literacy). Five subscales: informed, values clarity, support, uncertainty, and effective decision. (Some reviews may refer to the original 3 subscales only.) 16 items, 5-point Likert scale statement responses from 0 (*strongly agree or yes*) to 4 (*strongly disagree or no*).	The total score for items 1 to 16 is summed and converted to a score ranging from 0 (*no decisional conflict*) to 100 (*extremely high decisional conflict*). Low decisional conflict <25, increased decisional conflict >37.5.^27^	Informed subscale: “I know which items are available to me.”
Multidimensional Measure of Informed Choice (MMIC) Marteau et al. 2001,^20^	Informed choice “An informed choice is one that is based on relevant knowledge, consistent with the decision-maker’s values and behaviourally implemented.”^20^ Theory of Planned Behaviour^87^	Knowledge and value consistency (attitudes and screening behavior) combined into a single measure. 8-item knowledge questionnaire with multiple-choice questions. 4-item attitude scale with 7-point numerical response options to statement. Uptake determined by whether screening test undertaken yes/no.	Scores for each scale individually summed. Good knowledge cutoff median >4. Positive attitude cutoff median >22. Uptake of screening yes/no. An informed choice is made when an individual has good knowledge consistent and uptake consistent with their attitude.	Knowledge: “Which of these conditions do you think that the test screens for?”
Control Preferences Scale (CPS) Degner et al. 1997,^88^	Role preference in health care decision making “. . . the degree of control an individual wants to assume when decisions are being made about medical treatment.”^88^ Grounded and unfolding theory.^88^	Originally 5 playing cards portraying different roles consumers could assume in treatment decision making. Recent studies have modified this to a single-item 5-point scale representing their desired role. Participants make comparisons and produce their total preference order for the roles.	Order of roles ranging from most to least preferred. If single-item version is used, the point scale single answer is used. Preferences can then be analyzed using unfolding theory to estimate the degree of control desired in different populations/contexts.	Active role A: “I prefer to make decisions about which treatment I will receive.”
State-Trait Anxiety Inventory (STAI) Spielberger et al. 1970,^89^	Anxiety “Anxiety is perhaps most commonly used to denote a complex emotional reaction or state that varies in intensity and fluctuates over time as a function of the intrapsychic or situational stresses that impinge upon an individual.”^90^ Model distinguishing between trait anxiety (A-Trait) and state anxiety (A-State).^90^	40-item total scale consisting of 20-item A-State questionnaire and 20 item A-Trait questionnaire. Responses recorded on a 4-point scale from 1 (*not at all*) to 4 (*very much so*).	The two 20-item scales are scored individually with scores ranging from 20 to 80 for each. Higher scores indicate higher anxiety. Different cutoffs available. For some, ≥40 = clinical anxiety. Others group into no or low (20–37), moderate (38–44), and high (45–80) anxiety.	A-State item: “I feel comfortable.”
Quality of Informed Consent (QuIC) Joffe et al. 2001,^91^	Informed consent “Elements of informed consent include capacity, disclosure, understanding, voluntariness and permission.”^91^ Federal regulations governing research with human subjects,^92^ theoretical work by Appelbaum et al.,^93^ and the recommendations of National Cancer Institute’s working group.^94^	34-item scale divided into 2 parts: Part A – objective understanding (20 items) and Part B – subjective understanding (14 items). Part A response options in a 3-point Likert scale from 1 (*disagree*) to 3 (*agree*). Part B response options in 5-point Likert scale from 1 (*I didn’t understand this at all*) to 5 (*I understand this very well*).	Correct answers in Part A = 100 points, unsure = 50 points and incorrect/not answered = 0 points. Points across domains are averaged and converted to a score/100 overall. The average of scores in Part B (1–5) are converted to a score/100. Scores for Part A and Part B can be compared across studies.	Part A: “When I signed the consent form for my current cancer therapy, I knew that I was agreeing to participate in a clinical trial.”
Satisfaction with Decision (SwD)^a^ Barry et al.^95^	Satisfaction Not available Not available	3-item scale with 5-point Likert responses.^59^	Not available	Not available
Satisfaction with Decision-Making Process (SwDM)^a^ Barry et al.^95^	Satisfaction Not available Not available	12-item scale with 5-point Likert responses from 1 (*strongly agree*) to 5 (*strongly disagree*) for items 1 to 5 and 1 (*excellent*) to 5 (*poor*) for items 6 to 12^59,131^	Scores from the items summed and normalized to yield a score ranging from 0% to 100%.^131^	“I got as much information as I wanted about my heart condition.”^131^
Satisfaction with Decision Scale (SwDS) Holmes-Rovner et al. 1996,^96^	Satisfaction Not available Built on O’Connor and O’Brien-Pallas’s conceptual model of an effective decision (no theoretical framework stated).^28^	6-item scale with 5-point Likert responses from 1 (*strongly disagree*) to 5 (*strongly agree*).	Scored 1 to 5 with higher scores indicating higher satisfaction.	“I am satisfied that I am adequately informed about the issues important to my decision.”

a Unable to access the original article. Therefore, some information is unavailable and is instead taken from other articles using scale or reviews appraising scale.

**Table 12:** Psychometric Properties and Clinical Sensibilities of Common Measurement Instruments Identified in Systematic Reviews

Measurement Instrument	Reliability		Validity				Other
	Internal Consistency (Cronbach’s alpha)	Test-Retest	Face	Content	Construct	Criterion	
DCS	0.78–0.92^67,97^	0.81^67,97^	•^23,59,67^	•^21,59,67^	•^21,59,67,97^	•^21,59,97,98,99^	• Responsiveness^59,97^ • Feasibility^59,67^ • Acceptability^59,67^
MMIC	K: 0.68–0.82^20,100^ A: 0.78–0.83^20,100^				**•** ^20^	**•** ^100^	• Feasibility^20,27,100^ • Acceptability^20,27,100^
CPS	0.72^132^		•^59^		•^88,101^		• Precision^59^ • Responsiveness^59^ • Feasibility^59^ • Acceptability^59^
STAI	A-S: 0.83–0.95^90–103,105^ A-T: 0.82–0.92^90–103,105^	A-S: 0.16–0.63^90–103,105^ A-T: 0.73–0.86^90–103,105^		•^106^	•^90,107,108^	•^90,107^	• Feasibility^106,109^ • Acceptability^107^
QuIC		Part A: 0.66^91^ Part B: 0.77^91^	•^91^	•^91^			• Feasibility^91^ • Acceptability^91^
SwD^a^	0.81–0.85^95,110^		•^59,95^	•^59,95^			• Feasibility^59,95^
SwDM^a^	0.94–0.95^95,110^		•^59,95^				• Feasibility^59,95^
SwDS	0.86^96^				**•** ^96^	**•** ^96^	• Feasibility^96^ • Acceptability^96^

CPS, Control Preferences Scale; DCS, Decisional Conflict Scale; MMIC, Multidimensional Measure of Informed Choice; QuIC, Quality of Informed Consent; STAI, State-Trait Anxiety Inventory; SwD, Satisfaction with Decision; SwDM, Satisfaction with Decision-Making Process; SwDS, Satisfaction with Decision Scale.

a Unable to access the original article. Therefore, some information is unavailable and is instead taken from other articles using scale or reviews appraising scale.

### Decisional Conflict Scale (DCS)^67^

The DCS by O’Connor et al.^67,97^ was discussed most frequently across reviews^21,23,28,19,43,59,66^ and measures an individual’s uncertainty in decision making.^67^ Of the 4 existing versions, the statement format is the most commonly used in health care studies.^97^ The DCS was the most common validated measure in 4 reviews^19,43,59,66^ and the second most common in a fifth.^27^ Notably, the DCS was used as a measure of the decision process in more than 50% of studies in 2 reviews.^43,66^ As Table 12 demonstrates, there is a general agreement and evidence toward multiple facets of validity and other psychometric properties for the DCS, although occasional discrepancies do exist. For example, although Sepucha et al.^43^ found 15% of their studies reported on precision/accuracy, precision was the only area in which no data were available in the studies included in Kryworuchko et al.’s review.^59^

### Multidimensional Measure of Informed Choice (MMIC)^20^

The MMIC by Marteau et al.^20^ was the second most highlighted review measure.^27,43,62,63^ They adapted O’Connor et al.’s effective decision to define informed choice after suggesting that existing measures were not multidimensional, usually only assessing knowledge.^20,28^ Less widely validated than the DCS, as evidenced in Table 12, the MMIC was the most used measure in Ames et al.’s reproductive screening review in 17 of 33 studies.^27^ This review also thoroughly discussed the measure’s performance, with criticism including the dichotomization of knowledge and attitude scales relying on a cutoff, of which there are varying standards (e.g., for “good” knowledge).^27^ Additionally highlighted was the risk of oversimplification of choice through the use of binary variables and the lack of cognitive processes (e.g. deliberation included).^27^

### Control Preferences Scale (CPS)^88^

The CPS by Degner et al.^88^ aims to measure health care consumers’ desired level of control and was conceived during a period when evidence showed better outcomes for those involved in their treatment decisions. Reviews highlighted its popular use as a decision process measure and in evaluating role preference.^23,66^ One review specifically by Kryworuchko et al.^59^ found that although used in only 2 of 35 studies, it psychometrically scored the second highest after the DCS, as reflected in Table 12, and was particularly praised for its feasibility in subsequent studies.^59^

### State-Trait Anxiety Inventory (STAI)^90^

First conceptualized in the 1960s by Spielberger et al., the STAI is based on a model distinguishing between state anxiety, a transitory emotional response to perceived dangers (A-State), and trait anxiety, a stable disposition to respond anxiously to threats (A-Trait).^90–103^ The test–retest reliability values in Table 12 reflect A-State transience and are expectedly lower than A-Trait.^102,103^ Despite the debate on its utility,^39,111^ anxiety is commonly measured as a decision aid outcome reflecting the process or post–decision outcomes.^23,31,42,104^ Reference to its use by included studies was noted by multiple reviews, but it received minimal discussion,^23,19,64^ with Table 12 evidence an example supported by additional literature.^90–103,105–109^

### Quality of Informed Consent (QuIC)^91^

The QuIC questionnaire by Joffe et al.^91^ was developed to standardize consent evaluation and measures actual (objective) and perceived (subjective) understanding. The QuIC was featured in 3 reviews,^37,38,61^ although it was the most common measure in only one.^61^ It was particularly highlighted by Gillies et al.^37^ for assessing 4 of 5 domains of understanding in informed consent (content validity) and praised by Montalvo et al.^61^ and Sand et al.^38^ for its described development and validation compared with other instruments.

### Satisfaction Measures^95–96^

Satisfaction and decision regret scales measure decision outcomes through the hypothesis that informed decisions may increase satisfaction/decrease regret.^27^ Six reviews discussed satisfaction with the decision or decision-making process.^23,27,19,61,59,64^ Although other validated scales were mentioned,^112,113^ 3 were highlighted as used in subsequent papers following their initial development: Barry et al.’s Satisfaction with Decision (SwD),^95^ Barry et al.’s Satisfaction with Decision-Making Process (SwDM),^95^ and the Holmes-Rovner et al. Satisfaction with Decision Scale (SwDS).^96^

The SwD and SwDM scales feature across 4 reviews.^23,27,19,59^ The SwD assesses whether the right choice was made and satisfaction with the decision and rates the decision itself.^59,95^ The SwDM covers questions assessing information adequacy, support, and role satisfaction.^59,95^ Kryworuchko et al.’s^59^ review specifically appraised them, with both scoring positively for reliability, validity, and feasibility and the SwDM additionally scoring for responsiveness and interpretability. Holmes-Rovner et al.’s SwDS featured in trials in 4 reviews.^23,19,61,64^ Although its validity was acknowledged,^23,64^ the relevance of this measure in easily reversible decisions as opposed to more permanent decisions has been questioned.^23^

## Discussion

### Key Findings

Informed choice has become increasingly prominent in health care.^1–5,40^ Despite the spotlight and interventions strategized to improve it,^40,42^ this review highlights the persisting challenges in the operationalization and measurement of informed choice.

### Difficulties in Conceptualizing Informed Choice and Associated Concepts

Systematic reviews used varying terminology and definitions, reinforcing confusion and heterogeneity. While most reviews analyzing the same concept (e.g., informed consent) aligned broadly, no 2 definitions matched.

Informed consent definitions emphasized information provision, capacity, and voluntariness, reflected in the domains given in Table 7.^36–38,61,64,60,82^ Such an agreement likely reflects the strong ethical and legal foundations of informed consent shaped by Beauchamp and Childress and the WMA statements.^1,6,32^ Yet, alternative interpretations persist, such as Raper et al.’s distinct diagnostic approach,^64^ contributing to ongoing variation.

Similarly, although informed choice, informed decision making, effective decision, and decision quality and process are often considered as separate concepts, Tables 6, 8, and 9 highlight their commonalities.^23,27,29,19,43,62–63^ For example, O’Connor et al.’s definition of effective decision—centered on information and value congruence^28^—appeared across other terms.^15,20,27–31^ These overlaps suggest potential for conceptual bridging, as reflected in shared domains in Table 8.

### Lack of Theory and PPI Underlying Measurement Instrument Development

Reviews noted the lack of theory and PPI in developing measures, raising concerns about the instruments’ validity.^37^ Many decision-making theories, such as normative, descriptive, and prescriptive theories, exist,^31^ guiding instrument development and reducing confusion surrounding concepts.^63^ The most commonly used and validated instruments highlighted in Tables 11 and 12 were explicit in their theory, for example, the MMIC (theory of planned behavior,^20,87^ or the CPS (grounded and unfolding theories).^88^

### Heterogeneity and Lack of Standardization in Instruments

Measurement heterogeneity is the biggest challenge, noted in 12 systematic reviews,^v^ resulting in limited comparability across studies and a lack of meta-analyses.^vi^ The use of standardized instruments and adherence to consistent guidelines, such as those mentioned in the introduction, is encouraged.^vii^ Only 1 review, by Gillies et al.,^37^ followed COSMIN’s protocols,^44,45,114^ whereas 4 used IPDAS criteria.^29,43,59,66^ Despite available guidelines such as the ELICIT core outcome set developed by the authors of 1 included systematic review,^37,48^ this umbrella review highlights these ongoing measurement inconsistencies.

### Decision to Use Investigator-Developed Instruments and Inconsistent Instrument Validation

Researchers often chose unique, investigator-developed instruments,^36,38,61–59,60^ frequently lacking details on their development or validation,^viii^ rather than using preexisting scales.^ix^ Even the reporting of validated tools varies.^23,43,66^ Journal word limits may restrict details, although supplementary materials can alleviate this.^43^

### Characteristics of Measurement Instruments

The DCS, MMIC, CPS, STAI, QuIC, and satisfaction measures were among the most frequently used and well-appraised tools, with many having gained seminal status.^67,88,90^ These instruments are theory or framework based with varying psychometric properties, with some measuring entire concepts (e.g., MMIC or QuIC),^20,91^ while others focus on specific domains or subconcepts.^18,41,67,88,95–96^ To fully assess informed choice, multiple instruments are often needed.^21^ Even multidimensional tools such as the MMIC do not cover aspects like anxiety, regret, or satisfaction,^20^ although the utility of measuring some of these remains undetermined.^39,40^

### Recommendation Summary for Researchers

Table 13 summarizes the recommendations for researchers measuring informed choice in the future. While researchers will primarily be responsible for implementing these, the wider scientific community also has an important role. For example, in their critical appraisal, reviewers should consider whether measurement instruments have been validated with available results. Editors can consider population diversity across studies they are publishing, and organizations such as IPDAS should continue to provide up-to-date evidence-based guidance for researchers to access.^78^

**Table 13:** Recommendations for Consideration to Be Made When Measuring Informed Choice

Recommendations
• Researchers should develop and use consistent terminology, definitions, and conceptualization of the construct they are measuring, e.g., informed choice.
• Researchers should aim to widen the diversity of the populations in which these measurement instruments are developed and evaluated to better reflect the populations they serve in clinical practice.
• Where preexisting, multidimensional, well-validated (including less commonly reported psychometric properties) measurement instruments are available, researchers should aim to use these for consistency in the literature.
• Where the development of a new measurement instrument is required, researchers should aim to develop multidimensional instruments that extend beyond solely assessing knowledge that has been validated with their psychometric evidence available.
• When developing these measurement instruments, researchers should incorporate existing theoretical frameworks and patient and public involvement.

### Strengths

To our knowledge, this is the first umbrella review evaluating the operationalization and measurement of informed choice in health care, presenting a concise summary for health professionals, researchers, and policy makers. The methods and tools used follow Joanna Briggs Institute (JBI) and Cochrane recommendations, with a protocol available on Prospero to maintain transparency. A comprehensive search strategy in consultation with specialist librarians was performed, and study selection, appraisal, and data extraction were conducted in duplicate in alignment with guidelines.^115^ The ROBIS critical appraisal allows for the interpretation of results accordingly, and despite the potential bias identified through this, the highest evidence available has informed findings.

### Limitations

Despite following JBI, Cochrane, and PRIOR guidelines,^50,52,53^ limitations remain. Informed choice is defined in varied ways,^27,19,36,38,61,62,60^ complicating the search strategy. Terms were chosen after scoping and consultation but may not cover all indexing variations. The search was conducted only once, and to comply with PRIOR recommendations,^53^ only systematic reviews were included, meaning other review types that may have contained relevant data were excluded. Reviews were limited to adult populations with presumed competency in decision making from a patient perspective to enable comparison between reviews. The measurement of informed choice in other populations or from a professional or observer perspective is an important research question that this review cannot answer.

Conceptual overlaps, such as informed consent or informed decision making, added complexity.^11,15,20,27,38^ Eligibility criteria were carefully defined and justified under the methods section, although the inability to include possibly relatable concepts is recognized as a limitation. The ROBIS appraisal identified many reviews as high risk for bias, limiting confidence in conclusions. Despite these limitations, including all reviews regardless of their appraisal score appears to be the most appropriate method, where the purpose was to summarize the available evidence. Aside from not performing a risk-of-bias assessment themselves, most reviews otherwise scored well in the phase 2 questions. Furthermore, as one review discussed, the purpose was to assess methods (measurement instruments) rather than outcomes.^63^ If anything, this quality appraisal presents where improvements in the literature are required.

Limitations also arose from using tools designed for quantitative, interventional trials, necessitating flexibility with guidelines, including SPIDER and PRIOR.^53,116^ Given the heterogeneity of the included reviews, meta-analysis was not possible, with the conclusions drawn limited to those from narrative synthesis. This reflected the challenge faced by most of the included systematic reviews, with only 2 performing meta-analyses^36,62^ and others citing similar reasons for not doing so.^21,27,19,61,63,64^

### Bridging the Gap for the Future

This umbrella review examined how informed choice is measured in health care, highlighting challenges that require further research. Core outcome sets and guidelines, such as COSMIN, aim to streamline this process.^44,45,48,49,114^ Meanwhile, this review also reveals gaps that can be bridged across concepts and stakeholders—researchers, health care professionals, and policy makers alike.

Conceptual similarities are clear: knowledge is measured almost universally, and recommendations such as PPI in measure development and psychometric testing apply across concepts. While they may have originated in different contexts and diverged in their integration and acceptance into health care, there are examples of how advances in one area can inform another. For example, informed consent is a cemented concept in health care contexts such as surgery or clinical trials.^1–3^ Its importance is exemplified in research falling under the United States Food & Drug Administration guidelines^117^ and the UK General Medical Council Consent guidelines for practitioners.^118^ Conversely, in screening, the focus has only recently shifted from promoting uptake to informed choice.^15,119–122^ This change is reflected in the updated UK National Screening Committee guidance,^5^ in which many informed consent studies are cited, reflecting the influence of the informed consent literature on the program’s quality assurance evaluation.^123^

This review bridges research and practice, highlighting the relevance of findings beyond academia to other stakeholders. For policy makers, informed choice requires clear measurement and evaluation strategies. With limited resources^9^ and RCTs often unfeasible,^124–126^ efficient approaches using practice-based or policy-driven evidence are needed.^124–126^ Despite limitations in evidence quality, this umbrella review can be used by policy makers for practice. Health care professionals play a key role in facilitating informed choice at the clinical interface, and a familiarity with these concepts and measures supports reflection on clinical interactions. Presenting exemplar instruments offers a foundation for understanding perspectives and selecting the most appropriate tools.

Although improvements continue, what tools are currently accessible to users? The MMIC appears most suitable for measuring informed choice, assessing both knowledge and value congruence. Alternatively, combining the knowledge questionnaire, DCS, and the 4 MMIC value items enables the evaluation of decision quality and process. Promising tools include the Amsterdam Informed Decision-Making Scale, recently presented as a preliminary conference poster, which incorporates individual information needs and decision processes informed by theory and PPI.^127^

In clinical trials, key informed consent domains in competent patients include information provision, comprehension, and voluntariness.^1,2,6,21,48,64,69–76^ The QuIC instrument is well-regarded with supporting validity evidence.^37,38,61^ Alternatively, the Brief Informed Consent Evaluation Protocol (BICEP) is a multidimensional, validated measure that includes open-ended questions and satisfaction assessment.^128^ Other suitable domains or instruments likely exist beyond those identified in this review.

## Conclusion

This review emphasizes that although informed choice is crucial in health care, its measurement and operationalization remain debated. A first step is achieving consistent definitions of informed choice and related concepts. Following this, using standardized, validated, multidimensional instruments informed by theory and PPI in diverse populations is recommended. Available examples include the DCS, MMIC, and QuIC. While these future steps undergo implementation, this review provides a unified piece of work for health professionals, researchers, and policy makers to access to understand the different components of these concepts and choose the best-suited measurement instrument.

## Supplemental Material

sj-docx-1-mdm-10.1177_0272989X251413276 – Supplemental material for Operationalizing and Measuring Informed Choice in Health Care: An Umbrella Review Supplemental material, sj-docx-1-mdm-10.1177_0272989X251413276 for Operationalizing and Measuring Informed Choice in Health Care: An Umbrella Review by Holly Sprosen, Chiara Re, Grant D. Stewart and Juliet A. Usher-Smith in Medical Decision Making

## Notes

i. References 20, 22, 26, 29, 34, 36, 56, 60, 62.

ii. References 20, 29, 30, 34–36, 41, 56–60, 62.

iii. References 20, 26, 29, 30, 34–36, 41, 56–62.

iv. References 20, 22, 26, 29, 30, 34–36, 56, 57, 60, 62.

v. References 20, 22, 26, 29, 30, 34–36, 56, 57, 60, 62.

vi. References 26, 29, 30, 34–36, 41, 56–58, 61.

vii. References 20, 22, 26, 29, 34, 36, 56–58, 60.

viii. References 22, 26, 29, 34, 36, 41, 57, 59, 62.

ix. References 20, 22, 26, 30, 34, 36, 41, 56, 62.
