# Supplementary material for: Operationalizing and Measuring Informed Choice in Health Care: An Umbrella Review
Source: Med Decis Making. 2026 Mar 15;46(4):501–26. doi: 10.1177/0272989X251413276 (PMC13062464; doi:10.1177/0272989X251413276)
Supplement: sj-docx-1-mdm-10.1177_0272989X251413276 – Supplemental material for Operationalizing and Measuring Informed Choice in Health Care: An Umbrella Review [file sj-docx-1-mdm-10.1177_0272989X251413276.docx]

**Appendices**

**Appendix 1:** Example search strategy for Ovid EMBASE.

1. (healthcare or health-care or “health care” or care or medic* or surg* or treatment or screening or trial*).ti,ab
2. clinical trial/
3. public health/
4. surgery/ or medical procedures/
5. mass screening/ or cancer screening/ or prenatal screening/
6. 1 or 2 or 3 or 4 or 5
7. (informed adj (consent or choice* or decision* or participat*)).ti,ab.
8. (decision* adj3 (shared or making or regret or satisfact* or conflict)).ti,ab.
9. (voluntary adj3 (consent or choice* or decision*)).ti,ab.
10. (evidence adj3 (consent or choice* or decision*)).ti,ab.
11. (autonomous adj3 (consent or choice* or decision*)).ti,ab.
12. (preference adj3 decision*).ti,ab.
13. “patient participat*”.ti,ab.
14. informed consent/
15. decision making/ or patient decision making/ or shared decision making/
16. 7 or 8 or 9 or 10 or 11 or 12 or 13 or 14 or 15
17. (measure* or tool* or scale* or framework* or questionnaire*).ti,ab.
18. 6 and 16 and 17
19. limit 18 to (human and english language and (meta analysis or “systematic review”) and yr=”1990-Current”)

**Appendix 2:** Data extraction template converted from multi-page format.

| **Data to extract** |
| --- |
| 1. Title, Author, Year of Publication, Journal |
| 1. Primary or updated review |
| 1. Based on a Cochrane review yes/ no (details included if yes) |
| 1. Review context/ setting |
| 1. Aim/ objectives/ research question |
| 1. Concept assessed by review |
| 1. Definition for concept used by review |
| 1. Guidelines or theoretical frameworks referenced |
| 1. Number and type of individual studies included |
| 1. Analysis approach performed (with justification if reported) |
| 1. COSMIN tool used yes/ no |
| 1. Properties of measurement instruments assessed |
| 1. Key study findings |
| 1. Psychometrics performed/ appraised with results |
| 1. Meta-analysis details if performed |
| 1. Number of measurement instruments identified |
| 1. Most common/ appraised measurement instruments highlighted by reviews |
| 1. Findings related to these specific measurement instruments highlighted |
| 1. Weaknesses and limitations identified in the literature by reviews |
| 1. Recommendations made by reviews |

**Appendix 3:** Excluded studies with reason for not meeting eligibility criteria.

|  | Paper | Reason |
| --- | --- | --- |
| 1 | Aasen DM et al. 2020 | Measures preoperative provision of risk information by surgeons. Does not include a measure from the patient's perspective. |
| 2 | Ahmad M et al. 2020 | Appraises measurement instruments for shared decision-making. Does not measure a described concept. |
| 3 | Amaral AS et al. 2022 | Summarises measurement instruments for healthcare decision-making capacity. |
| 4 | Auerbach SM 2001 | Literature review on desire for control in decision-making. Not a systematic review. |
| 5 | Austvoll-Dahlgren A et al. 2016 | Mapping review of interventions and assessment tools for concepts appraising treatment effect claims. Not a systematic review. |
| 6 | Bekker HL et al. 2003 | Appraises anxiety measurement instrument for decision aid effectiveness. Does not measure a described concept. |
| 7 | Boivin A et al. 2018 | Appraises evaluation tools for patient and public engagement in research and decision-making. Does not measure a described concept. |
| 8 | Bouniols N et al. 2016 | Appraised measurements for shared decision-making that can be adapted in French context. Does not measure a described concept. |
| 9 | Chewning B et al. 2012 | Evaluates decision role preferences of patients in treatment and screening setting. Does not measure a described concept. |
| 10 | Convie LJ et al. 2020 | Describes the experiences, concerns and needs of patients and professionals for informed consent. Does not evaluate measurement instruments. |
| 11 | Convie LJ et al. 2018 | Describes a protocol for the development of a core outcome set for informed consent. Not a systematic review. |
| 12 | Couët N et al.2015 | Appraises the OPTION instrument measuring the extent to which healthcare providers involve patients in decision-making. Does not include a measure from the patient's perspective. |
| 13 | De Mik SML et al. 2018 | Evaluates shared decision-making metrics and outcomes in surgery. Does not measure a described concept. |
| 14 | Doherr H et al. 2017 | Evaluates the use of the Shared Decision-Making Questionnaire in intervention studies. Does not measure a described concept. |
| 15 | Dy SM 2007 | Structured literature review of measurement instruments evaluating shared decision-making. Not a systematic review. |
| 16 | Edwards A et al. 1999 | Reviews outcomes used in risk-communication studies. Does not measure a described concept. |
| 17 | Edwards SJL et al. 1998 | Examines the effectiveness of different methods of obtaining informed consent. Does not evaluate measurement instruments. |
| 18 | Elwyn G 2003 | Describes the development and validation of the OPTION tool measuring the extent to which healthcare providers involve patients in decision-making. Does not include a measure from the patient's perspective. |
| 19 | Elwyn G et al. 2001 | Appraises measurement instruments evaluating the extent professionals involve patients in decision-making from observer perspective. Does not include a measure from the patient's perspective. |
| 20 | Forshaw KL et al. 2016 | Evaluates instruments measuring patient's preparedness for medical evaluations. Does not measure a described concept. |
| 21 | Gärtner FR et al. 2018 | Protocol for the development of a core outcome set for the evaluation of interventions that improve informed consent. Not a systematic review. |
| 22 | Gillies K et al. 2015 | Describes the development of a core outcome set for the evaluation of interventions aiming to improve informed consent. Does not evaluate measurement instruments. |
| 23 | Gillies K et al. 2021 | Reviewed the process of obtaining informed consent in cluster randomised control trials. Does not evaluate measurement instruments. |
| 24 | Giraudeau B et al. 2012 | Appraises the quality of instruments assessing shared decision-making. Does not measure a described concept. |
| 25 | Hudon C et al. 2011 | Appraised instruments measuring patients' perception of patient-centred care. Does not measure a described concept. |
| 26 | Ignatowicz A et al. 2023 | Reviews interventions that improve ethical decision-making from a healthcare professionals perspective. Does not include a measure from the patient's perspective. |
| 27 | Ittenbach RF et al. 2023 | Trial evaluating a new measure of understanding of informed consent. Not a systematic review. |
| 28 | Jerofke-Owen T et al. 2020 | Appraises instruments measuring a patient's preference for engagement in decision-making. Does not measure a described concept. |
| 29 | Kylén M et al. 2022 | Scoping review instruments measuring patient participation in person-centred care. Not a systematic review. |
| 30 | Loosman I et al. 2022 | Analyses informed consent models. Does not evaluate measurement instruments. |
| 31 | Mavis B et al. 2015 | Appraises instruments measuring patient participation in healthcare encounters. Does not measure a described concept. |
| 32 | Merlo G et al. 2020 | Assesses discrete choice experiments in healthcare professional decision-making. Does not include a measure from the patient's perspective. |
| 33 | Naye F et al. 2024 | Scoping review of shared decision-making instruments for the development of a core outcome set in rheumatic and musculoskeletal care. Not a systematic review. |
| 34 | Niburski K et al. 2020 | Evaluates whether shared decision-making benefits surgical patients. Does not evaluate measurement instruments. |
| 35 | Norful AA et al. 2020 | Evaluated shared decision-making measurement instruments in chronic ambulatory care. Does not measure a described concept. |
| 36 | Normahani P et al. 2020 | Literature review of informed consent processes for the development of a informed consent framework. Not a systematic review. |
| 37 | Phillips NM et al. 2014 | Protocol for measuring patient participation in healthcare. Not a systematic review. |
| 38 | Phillips NM et al. 2016 | Appraises patient participation in healthcare measurement instruments. Does not measure a described concept. |
| 39 | Rathert C et al. 2022 | Conceptualises and reviews existing measures of therapeutic connection. Does not measure a described concept. |
| 40 | Russo S et al. 2019 | Review appraises psychological preferences and factors behind healthcare decision-making. Does not measure a described concept. |
| 41 | Saunders C et al. 2016 | Appraises instruments available that measure the cancer patient experience of healthcare. Does not measure a described concept. |
| 42 | Shay LA et al. 2015 | Evaluates shared decision-making measurements and patient outcomes. Does not measure a described concept. |
| 43 | Sugarman J et al. 1998 | Collated evidence on informed consent in literature and its challenges. Does not evaluate measurement instruments. |
| 44 | Toft BS et al. 2022 | Appraises measures assessing interventions aimed to increase patient involvement in healthcare. Does not measure a described concept. |
| 45 | Valentine KD et al. 2021 | Evaluation of the Shared Decision Making Process Scale through its use in published literature. Does not measure a described concept. |
| 46 | Vo H et al. 2023 | Secondary analysis of existing literature to determine the validity and reliability of the Shared Decision Making Process Scale. Not a systematic review. |
| 47 | Williams CJ et al. 1994 | Assesses the standard of informed consent in a trial. Not a systematic review. |

**Appendix 4:** ROBIS results per individual review.

|  | **Phase 2** | | | | **Phase 3** |
| --- | --- | --- | --- | --- | --- |
| **Review** | **1. Study eligibility criteria** | **2. Identification and selection of studies** | **3. Data collection and study appraisal** | **4. Synthesis and findings** | **Risk of bias in the review** |
| Afolabi et al. | 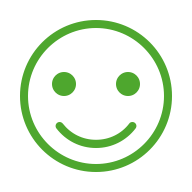 | 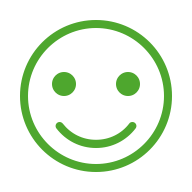 | 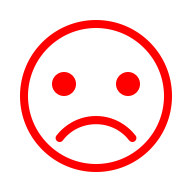 | 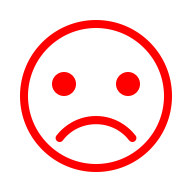 | 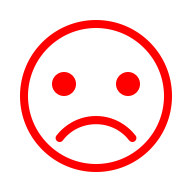 |
| Ames et al. | 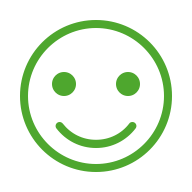 | 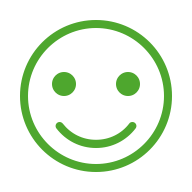 | 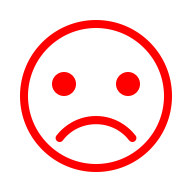 | 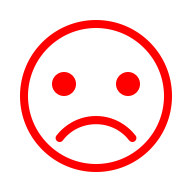 | 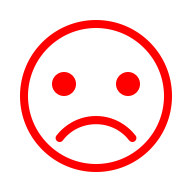 |
| Gillies et al. | 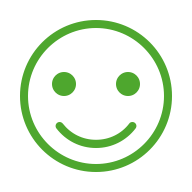 | 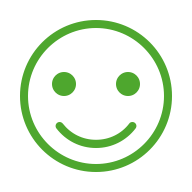 | 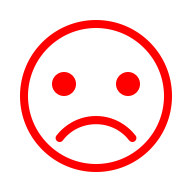 | 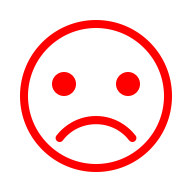 | 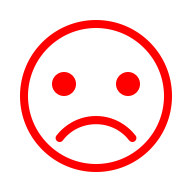 |
| Kennedy | 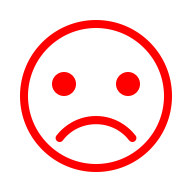 | 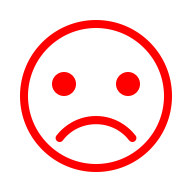 | 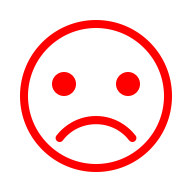 | 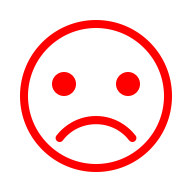 | 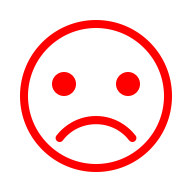 |
| Kryworuchko et al. | 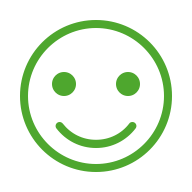 | 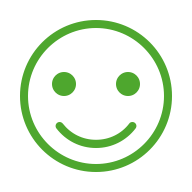 | 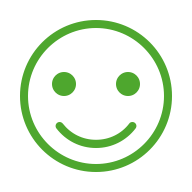 | 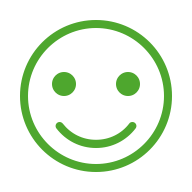 | 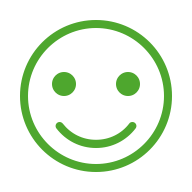 |
| Mamotte et al. | 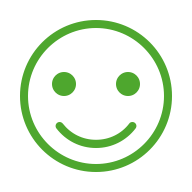 | 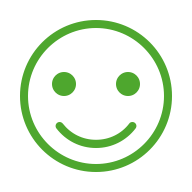 | 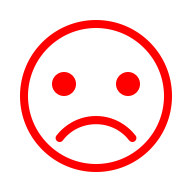 | 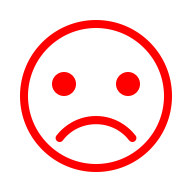 | 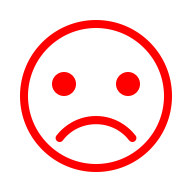 |
| Montalvo et al. | 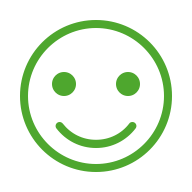 | 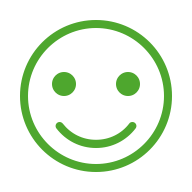 | 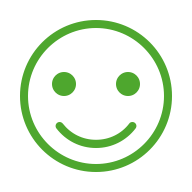 | 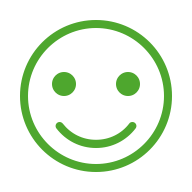 | 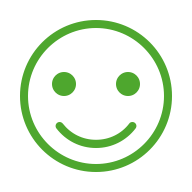 |
| Mullen et al. | 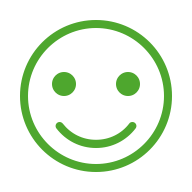 | 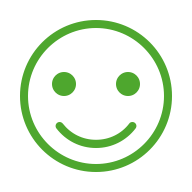 | 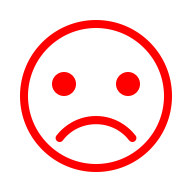 | 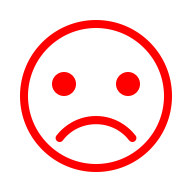 | 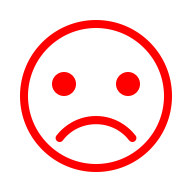 |
| Munro et al. | 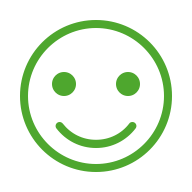 | 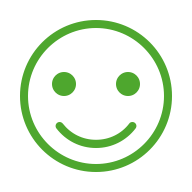 | 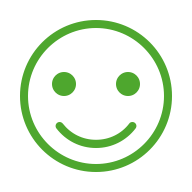 | 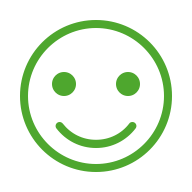 | 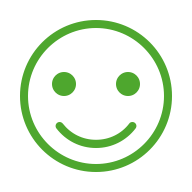 |
| Raper et al. | 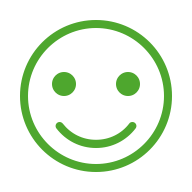 | 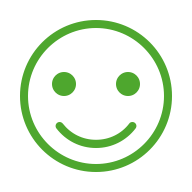 | 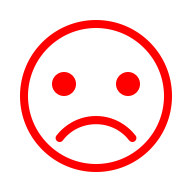 | 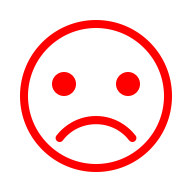 | 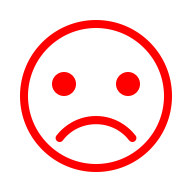 |
| Sand et al. | 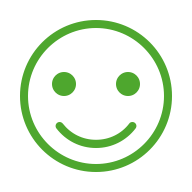 | 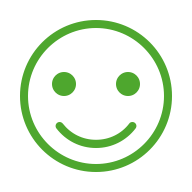 | 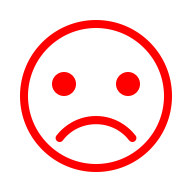 | 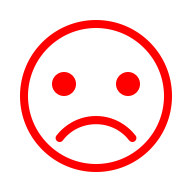 | 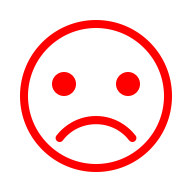 |
| Sepucha et al. | 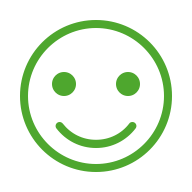 | 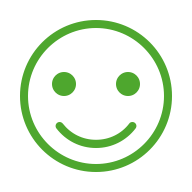 | 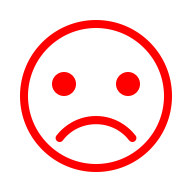 | 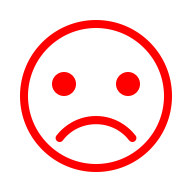 | 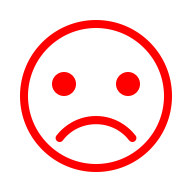 |
| Sepucha et al. | 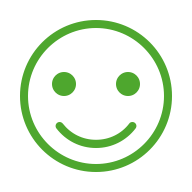 | 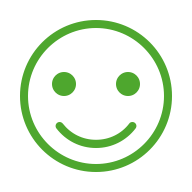 | 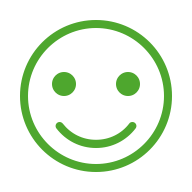 | 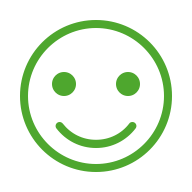 | 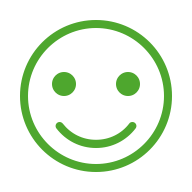 |
| Sherman et al. | 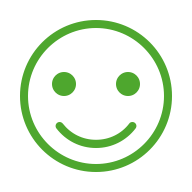 | 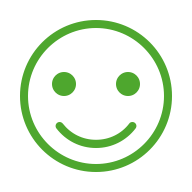 | 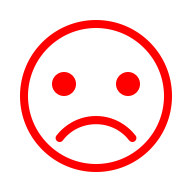 | 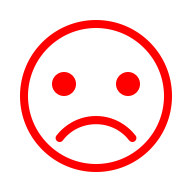 | 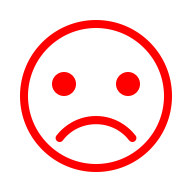 |
| Trenaman et al. | 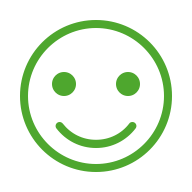 | 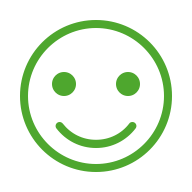 | 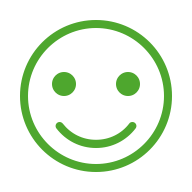 | 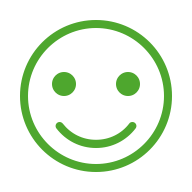 | 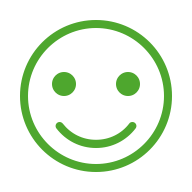 |
| Winn et al. | 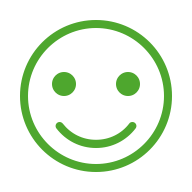 | 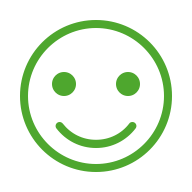 | 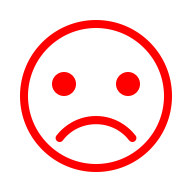 | 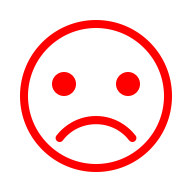 | 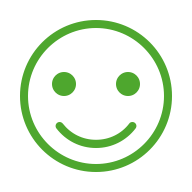 |

Key:
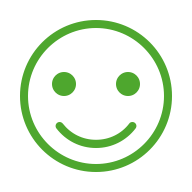
 = Low risk;
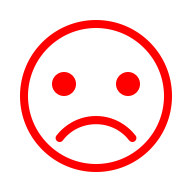
 = High risk;
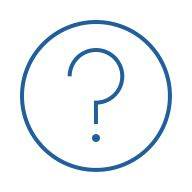
 = Unclear risk.

**Appendix 5:** Percentage of reviews attaining low, high or unclear risk of bias for each Phase 2 domain and overall in Phase 3.
